# Effects of Sigma-1 Receptor Ligands on Peripheral Nerve Regeneration

**DOI:** 10.3390/cells11071083

**Published:** 2022-03-23

**Authors:** Patrick Cottilli, Núria Gaja-Capdevila, Xavier Navarro

**Affiliations:** 1 Department of Cell Biology, Physiology and Immunology, Institute of Neurosciences, Universitat Autònoma de Barcelona, 01893 Bellaterra, Spain; cottillipatrick@gmail.com (P.C.); nuriagaja@gmail.com (N.G.-C.); 2 Centro de Investigación Biomédica en Red sobre Enfermedades Neurodegenerativas (CIBERNED), 28031 Madrid, Spain

**Keywords:** peripheral nerve injury, sigma-1 receptor, PRE-084, BD1063, Sig-1R KO, electrophysiology, nociception, intra-epidermal nerve fibers, myelinated axons regeneration, macrophage

## Abstract

Peripheral nerve injuries lead to the loss of motor, sensory and autonomic functions in the territories supplied by the injured nerve. Currently, nerve injuries are managed by surgical repair procedures, and there are no effective drugs in the clinic for improving the capacity of axonal regeneration. Sigma-1 receptor (Sig-1R) is an endoplasmic reticulum chaperon protein involved in many functions, including neuroprotection and neuroplasticity. A few previous studies using Sig-1R ligands reported results that suggest this receptor as a putative target to enhance regeneration. The aim of this study was to evaluate the possible effects of Sig-1R ligands on axonal regeneration in a sciatic nerve section and repair model in mice. To this end, mice were treated either with the Sig-1R agonist PRE-084 or the antagonist BD1063, and a Sig-1R knock-out (KO) mice group was also studied. The electrophysiological and histological data showed that treatment with Sig-1R ligands, or the lack of this protein, did not markedly modify the process of axonal regeneration and target reinnervation after sciatic nerve injury. Nevertheless, the nociceptive tests provided results indicating a role of Sig-1R in sensory perception after nerve injury, and immunohistochemical labeling indicated a regulatory role in inflammatory cell infiltration in the injured nerve.

## 1. Introduction

Peripheral nerve injuries (PNIs) produce partial or total loss of motor, sensory and autonomic functions of the affected parts of the body. This loss is caused by the interruption of axonal continuity, the degeneration of nerve fibers distal to the injury and eventually the death of the axotomized neurons. Although injured axons of the peripheral nerve are able to regenerate and reinnervate target organs, functional recovery is usually poor after severe nerve injuries. PNIs derived from a complete transection are managed surgically, in order to re-establish the continuity of the nerve. The gold standard for repairing small gaps is the direct suture of the disconnected nerve ends. However, the normal rate of axonal regeneration is only 1–2 mm/day [1], presenting an obstacle for the maintenance of the denervated parts. Thus, research on compounds that may accelerate axonal growth and target reinnervation will be relevant for reducing the consequences of chronic denervation (muscle atrophy, loss of sensory receptors) and improving recovery.

The sigma-1 receptor (Sig-1R) is an endoplasmic reticulum (ER) chaperone protein, mainly located at the mitochondrion-associated ER membrane (MAM). This receptor is expressed in several tissues, including neurons and glial cells of the nervous system [2]. In physiological states, Sig-1R is bound to the binding immunoglobulin protein (BiP). Upon ER stress or agonist activation Sig-1R dissociates from BiP and, among other functions, the receptor interacts with inositol 1,4,5-triphosphate receptor 3 (IP3R3), stabilizing its coupling with the voltage-dependent anion channel 1 (VDAC1), leading to increased Ca^2+^ flux from ER to the mitochondria [3,4]. This entrance regulates Ca^2+^ sensitive enzymes which are involved in ATP synthesis [5]. On the other hand, antagonist binding inhibits the aforementioned dissociation, impeding Sig-1R activity [6]. Thus, Sig-1R has an important role in Ca^2+^ homeostasis and ATP production, both required in energy demanding processes such as axonal regeneration. In fact, treatment with Sig-1R agonists PRE-084 and SA4503 has been shown to promote neurite elongation of cultured hippocampal neurons, whereas co-administration of a specific Sig-1R antagonist, NE-100, abolished the observed effects [7]. Similarly, PRE-084 treatment on organotypic spinal cord slices and dorsal root ganglia explants increased neuritogenesis, which was reverted by co-administration of the antagonist BD1063. Moreover, the effect was blocked when PRE-084 was administered together with chelerythrine, a protein kinase C (PKC) pan-inhibitor, suggesting the involvement of PKC in neuritogenesis [8]. The selective serotonin reuptake inhibitors, fluvoxamine and sertraline, enhance and reduce, respectively, the nerve growth factor-induced neurite outgrowth in PC12 cells in vitro. These effects are related to Sig-1R mediated mechanisms, since they were modified by addition of the agonist PRE-084 and the antagonist NE-100 [9,10]. Taken together, these results suggest the implication of Sig-1R in neuritogenesis of motor and sensory neurons. Surprisingly, Tsai and Su [11] observed axonal elongation with BD1063 treatment in primary cortical neurons, even though most of the literature about BD1063 is focused on neuropathic pain [12,13,14]. A wide range of evidence is now available to support the role of Sig-1R in the treatment of pain, mediating analgesia by Sig-1R antagonists [15], including models of neuropathic pain induced by partial nerve lesions [16], chronic constriction [14], diabetes [17] and chemotherapy administration [13]. Furthermore, the antagonist S1RA has shown promising results in a phase II clinical trial for neuropathic pain [18]. The potential mechanisms of action reported for the analgesic effects of Sig-1R antagonist ligands include mediation of opioid analgesia [19], a modulatory role in spinal sensitization through a Ca^2+^-dependent cascade [20] and regulating the recruitment of macrophages/monocytes into the injured nerve and dorsal root ganglia (DRG) [21].

This study aims to investigate the potential enhancement of axonal regeneration, modulation of neuropathic pain and inflammatory response after injury, by administration of Sig-1R agonist and antagonist compounds, in a mouse model of nerve transection and suture repair. To our knowledge, this is the first study addressing the effects of Sig-1R ligands on axonal regeneration in vivo.

## 2. Materials and Methods

### 2.1. Animals and Surgical Procedures

All the experimental procedures were approved by the Ethics Committee of the Universitat Autònoma de Barcelona (UAB). Mice were maintained in standard conditions with access to food and water ad libitum at the Animal Service of the UAB and were cared for and handled in accordance with the guidelines of the European Union Council (Directive 2010/63/EU). Thirty-one wild-type (WT) adult (10 weeks old) female C57BL/6 mice (Janvier Labs, Saint-Berthevin Cedex, France) were used in this study. Adult transgenic Sig-1R knock-out (Sig1R^−/−^) mice (10 weeks old) with C57BL6 background [2], were provided by Esteve Pharmaceuticals from the colony maintained at Envigo.

**Figure 1:**
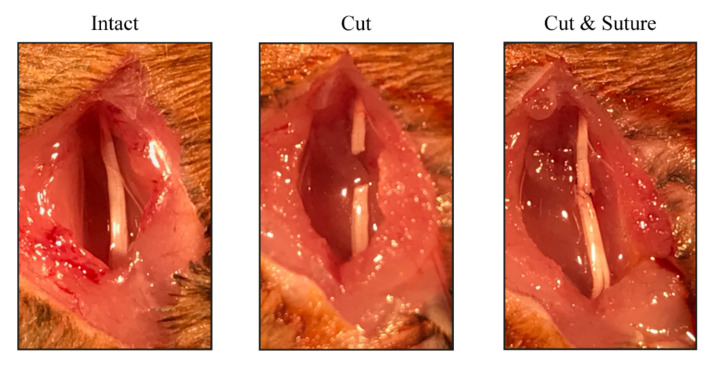
Illustration of the cut-and-suture surgical approach. The right sciatic nerve was exposed, cut at 43 mm from the tip of the 3rd toe and repaired by epineurial sutures.

To perform the nerve injury, mice were anesthetized with ketamine-xylazine (100–10 mg/kg). The right sciatic nerve was surgically exposed at the midthigh and cut at 43 mm from the tip of the 3rd toe and immediately repaired using two epineurial sutures (10–0), maintaining the fascicular alignment of the sciatic branches (Figure 1). The wound was sutured in planes and disinfected. Animals were left to recover on a hot pad after being returned to their cages. The left hindlimb was used as uninjured control. Mice were distributed in the following experimental groups depending on the treatment: saline (n = 12), PRE-084 (n = 8) and BD1063 (n = 8). For the Sig-1R ablation study, Sig-1R KO mice (n = 8) were followed up together with WT mice (n = 4). Animals were intraperitoneally administered either with PRE-084 (0.25 mg/kg, Tocris, Bristol, UK), BD1063 (5 mg/kg, Tocris) or saline serum (vehicle), 5 times per week for 42 days starting on the day of the surgery. The dose regime of the Sig-1R ligands was chosen based on previous reports indicating beneficial effects on spinal root injuries [22,23], pain models [24], and motoneuron preservation [25]. For analyses, the saline and WT groups were combined and named as control group.

### 2.2. Walking Track

**Figure 2:**
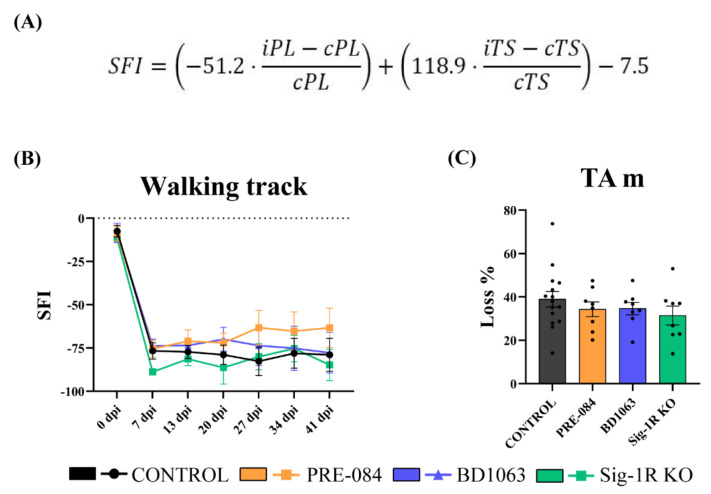
Functional evaluation after injury. (**A**) Sciatic functional index (SFI) is a measure of the pattern of hind paw stepping during locomotion, and requires an appropriate reinnervation of paw flexor/extensor muscles of the ankle and the toes after sciatic nerve injury. (i: ipsilateral paw; c: contralateral paw; PL: print length; TS: toe spread between 1st and 5th toes); (**B**) Plot of the walking track-derived SFI of the different groups of mice with Sig-1R ligands, and Sig-1R KO. Values at 0 dpi correspond to basal values from the test performed before the injury; (**C**) Percentage of weight loss of the TA muscle ipsilateral to injury with respect to the contralateral muscle at 42 dpi. Values are mean ± SEM; PRE-084 (n = 8), BD1063 (n = 8), Sig-1R KO group (n = 8) and control group (n = 15).

The walking track test was carried out prior to surgery to obtain baseline scores, and once per week after the surgery to assess the recovery of locomotor function. The plantar surface of the hind paws was painted with ink, and the animals were left walking on a sheet of white paper along a narrow corridor towards a dark compartment. The print length (PL) and the toe spread (TS) between 1st and 5th toes of each hindlimb were measured to calculate the sciatic functional index (SFI) (Figure 2A) [26]. For each animal and time point, three footprints were measured for both paws.

### 2.3. Electrophysiological Tests

Electrophysiological tests were performed at 14-, 21-, 28-, 35- and 42-days post-injury (dpi). Baseline values were obtained from the contralateral hind limb during the follow-up. Animals were anesthetized with pentobarbital (50 mg/kg i.p.), and placed prone on a thermostatic heating pad. The sciatic nerve was electrically stimulated with percutaneous needle electrodes placed at the sciatic notch. The compound muscle action potential (CMAP) of tibialis anterior (TA m) and plantar (PL m) muscles was recorded by microneedle electrodes placed on the muscle belly and the reference electrode at the tip of the fourth toe. In addition, the compound nerve action potential (CNAP) of the digital nerve (Dg n) was recorded with electrodes placed at the fourth toe. Action potentials were amplified and displayed on a digital oscilloscope (Tektronix TD420S, Tektronix, Wilsonville, OR) at appropriate settings to measure the latency and amplitude. For analysis, when no potential was recorded, the amplitude was considered as 0 mV, and the latency was given an arbitrary value of 50 ms.

### 2.4. Pinprick Test

The pinprick test was performed to assess the progression of nociceptive reinnervation at the hind paw [27]. Awake animals were lightly restrained and tested by pricking the skin surface of the lateral footpads and the fourth toe with a blunt needle to avoid skin damage. The animals were pricked three times at each site and given a score. When no response was observed the set value was 0, when inconsistent or reduced response the value assigned was 1, and when the response was comparable to the contralateral limb the value was 2. A global score to pinprick was constructed by the sum of the observed responses at the five tested sites.

### 2.5. Pincher Test

To determine the pain sensitivity, the Pincher test (IITC Inc., Woodland Hills, CA) was performed at 29 and 37 dpi. Basal values were obtained before the injury (0 dpi). Mice were gently immobilized in a home-made plastic cylinder and the hind paw subjected to an increased mechanical force in the middle of the plantar surface until a withdrawal response was produced. The maximum force applied was 260 g to avoid damage.

### 2.6. Immunohistochemical Analyses

At 42 dpi, mice were euthanized with pentobarbital, and tissue samples were harvested. Contralateral and ipsilateral tibial nerves above the ankle and footpads were harvested, fixed for 1 h in 4% paraformaldehyde in phosphate buffer (PB) and cryopreserved in sucrose 30% dissolved in PBS at 4 °C. In addition, the TA muscles of both hindlimbs were weighed to calculate the percentage of weight loss between the contralateral and ipsilateral muscles.

For the intra-epidermal nerve fiber (IENF) assessment, the C footpads were cut in 60 µm thick sections on a cryostat. Four central sections of each footpad were permeabilized with PBS 0.1 M with Triton X-100 0.3% (PBST) and incubated with blocking solution (PBST + Normal Donkey Serum (NDS) 1.5%) for 1 h. Then, sections were incubated free-floating with primary antibody anti-Protein Gene Product 9.5 (PGP9.5) (1:500; CL7756AP, Cedarlane) overnight at 4 °C. After washes with PBST, sections were incubated with secondary antibody conjugated to Cy3 (1:500; AP182C, Millipore, USA). Nuclei were stained with DAPI (1:2000; D9564, Sigma). The sections were dehydrated, mounted on gelatin-coated slides with DPX (06522, Sigma-Aldrich, USA) and viewed with an epifluorescence microscope (Olympus BX51, Olympus Europa, Hamburg, Germany). The number of nerve fibers inside the epidermis was counted from four sections per animal and expressed as number of IENF per mm skin length.

For immunolabeling macrophages, the tibial nerve was cut in 15 µm thick transverse sections and collected on gelatin-coated slides. The sections were washed, permeabilized with PBST, incubated with blocking solution for 1 h, and incubated with primary antibody anti-ionized calcium-binding adapter molecule 1 (IBA1) (1:500; 019-19741, Wako, Japan) overnight at 4 °C. After washes with PBST, sections were incubated with secondary antibody Alexa Fluor 594 donkey anti-rabbit IgG (1:500, A21207, Invitrogen, USA). The slides were washed and mounted with Fluoromount-G (0100-20, SouthernBiotech, USA) containing DAPI. Images of tibial nerve sections were captured with an epifluorescence microscope (Nikon ECLIPSE Ni), and the number of macrophages was counted using a macro built in ImageJ.

### 2.7. Histological Assessment of Regenerated Myelinated Axons

To assess the number of regenerated myelinated fibers, a segment of the distal part of the tibial nerve was postfixed in 3% paraformaldehyde—3% glutaraldehyde in PB. The nerves were postfixed and stained with 2% osmium tetroxide in PB for 2 h. The nerves were then washed, dehydrated with ethanol series and embedded in EPON resin (EMBed-812 EMBEDDING KIT #14120, Electron Microscopy Sciences, USA). Semithin sections (0.5 µm thickness) were cut with an ultramicrotome, collected on slides, and stained with toluidine blue. Myelinated axons were counted using ImageJ (Fiji software, https://fiji.sc/) [28] on microphotographs taken with a light microscope (Olympus BX40) attached to a digital camera (Olympus DP50) at 1000× magnification. At least 30% of the nerve cross-section area was analyzed to obtain a representative population of myelinated axons and calculate the estimated density. The total number of regenerated axons per nerve was calculated by multiplying the endoneurial area and the calculated density.

### 2.8. Statistical Analyses

Data were expressed as group mean ± SEM and analyzed with GraphPad Prism v8.0.2 (GraphPad Software). After assessing the homogeneity of variance with Bartlett’s, Levene’s (R 4.0.3) and Spearman’s tests between the experimental groups, statistical comparisons were made by two-way ANOVA followed by post-hoc Bonferroni test for multiple comparisons between groups. The results of the pinprick start day were compared using the non-parametric Friedman test. Differences were considered statistically significant at *p* value < 0.05.

## 3. Results

### 3.1. Functional Motor Recovery

The results of the walking track test showed that the SFI declined immediately after the nerve lesion and remained abnormal during the follow-up (Figure 2B), as expected after a neurotmesis injury [19]. A slight tendency to recover the SFI can be appreciated in the PRE-084 treated group from 27 dpi compared with the control and the BD1063 treated groups, although differences were not statistically significant. On the other hand, the Sig-1R KO group presented a similar pattern as the control and BD1063 groups.

At 42 dpi, the TA muscles were weighted, and the ratio between the lesioned and intact limbs was represented as percentage of weight loss. No statistical difference was observed between the mice groups (Figure 2C).

### 3.2. Electrophysiological Results of Nerve Regeneration after Sciatic Nerve Section and Repair

**Figure 3:**
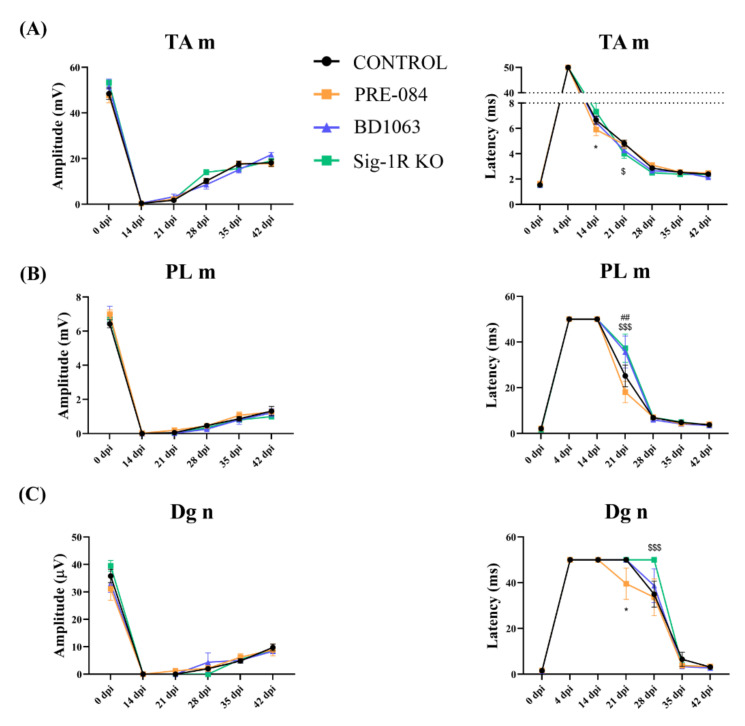
Electrophysiological results. Plots of CMAP amplitude and latency recorded from TA (**A**); and PL (**B**) muscles; and of CNAP amplitude and latency of the digital nerve (**C**) during the follow-up in the studies of Sig-1R ligands and Sig-1R KO mice. Values are mean ± SEM. Two-way ANOVA and Bonferroni post-hoc test: * *p* < 0.05 between PRE-084 and control group; ## *p* < 0.01 between BD1063 and control group; $ *p* < 0.05, $$$ *p* < 0.001 between Sig-1R KO and control group.

In order to study the progression of regenerating axons after the sciatic nerve injury, motor and sensory nerve conduction tests were performed repeatedly during the follow-up. At 14 dpi, CMAPs were absent on the two muscles tested, indicating complete denervation. At 21 dpi, reinnervation had started as indicated by recordings of small amplitude CMAP in the TA muscle of all mice, and in the PL muscle of some animals in all of the groups, with a higher proportion in the PRE-084 treated one (PRE-084 7/8–87.5%, BD1063 3/8–37.5%, Sig-1R KO 3/8–37.5%, control 10/15–66.67%). The amplitude of the CMAPs increased progressively at later time points, indicating progressive reinnervation of the muscles by the regenerated axons. The course of reinnervation of the TA muscle was comparatively faster than in the PL muscle, reflecting its more proximal location to the injury. Nevertheless, there were no significant differences regarding the CMAP amplitude evolution between treated and control groups (Figure 3A,B).

Regarding the sensory CNAP of the digital nerve, despite the fact that no differences were found between groups, the PRE-084 treated group presented an earlier response at 21 dpi, correlating with CMAP results (PRE-084 2/8–25%, BD1063 0/8, Sig-1R KO 0/8, control 0/15). In addition, Sig-1R KO mice started to have recordable CNAP one week later, at 35 dpi, than all the other groups, suggesting slower regeneration in the absence of the Sig-1R (Figure 3C).

For the latency of the CMAP and CNAP, some differences were observed between groups at early times of reinnervation, i.e., 14–21 dpi for the TA muscle (Figure 3A), 21 dpi for the PL muscle (Figure 3B) and 21–28 dpi for the digital nerve (Figure 3C). Interestingly, the PRE-084 treated group showed a shorter latency at 14 and 21 dpi, suggesting that at earlier times Sig-1R activation might increase myelination of the regenerating axons. On the other hand, the BD1063 treated group showed similar results than the Sig-1R KO group, with longer latencies than the control group.

### 3.3. Treatment with Sig-1R Antagonist BD1063 Produces Hyposensitivity after Nerve Injury

**Figure 4:**
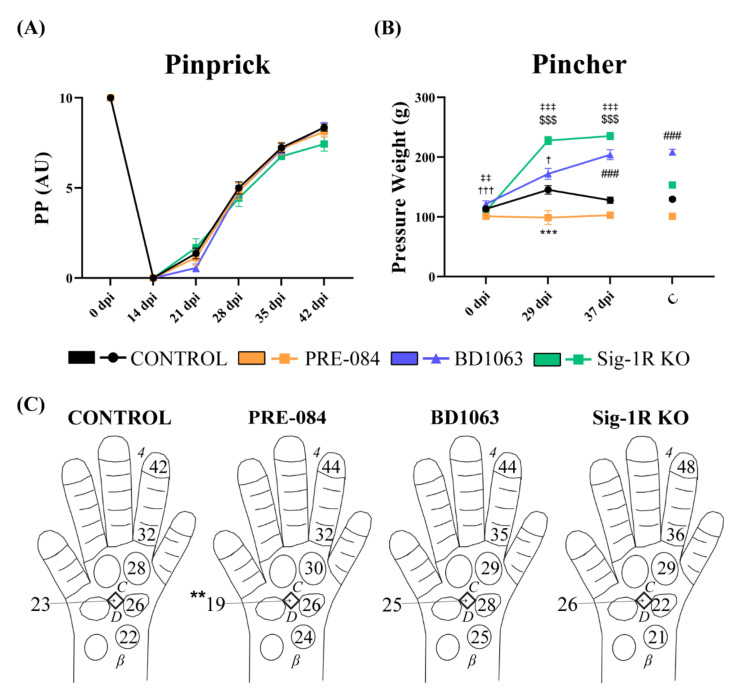
Nociceptive recovery. (**A**) Graph of the PP score during the follow-up; (**B**) Plot of the mean pressure weight, calculated in g, needed to evoke a withdrawal response with the Pincher test. *** *p* < 0.001 PRE-084 vs. control group; ### *p* < 0.001 BD1063 vs. control group; $$$ *p* < 0.001 Sig-1 R KO vs. control group; † *p* < 0.05, ††† *p* < 0.001 between injured and contralateral intact paw in BD1063 group (**C**); ‡‡ *p* < 0.01, ‡‡‡ *p* < 0.001 between injured and contralateral intact paw in Sig-1R KO group; (**C**) Representation of the mean day at which the PP response, and the PL m CMAP reappeared at the site tested in each group. ** *p* < 0.01 PRE-084 vs. BD1063 and Sig-1R KO groups.

Recovery of nociceptive sensitivity was assessed by the pinprick test. At 14 dpi there were no responses to pinprick stimulation in the denervated hind paw, therefore the PP score was 0. During follow-up, injured mice showed recovery of the sensory response but without differences between the groups (Figure 4A). The Pincher test was completed once PP responses had reappeared. The BD1063 treated group presented a significant hyposensitivity than the control group (Figure 4B), that was also observed in the contralateral limb. Conversely, the PRE-084 treated group showed a tendency to hyperalgesia in the ipsilateral paw, confirming the contrary effect to the antagonist treatment regarding pain sensibility. On the other hand, the Sig-1R KO group had a similar hyposensitivity as the BD1063 treated group.

Figure 4C shows a graphical representation of the starting day at which the PP response at each tested area of the denervated paw, and the PL CMAP were found. For the PP test, the responses started from proximal to distal areas, without consistent significant differences between groups.

### 3.4. Skin Reinnervation and Number of Regenerated Axons after Nerve Injury

**Figure 5:**
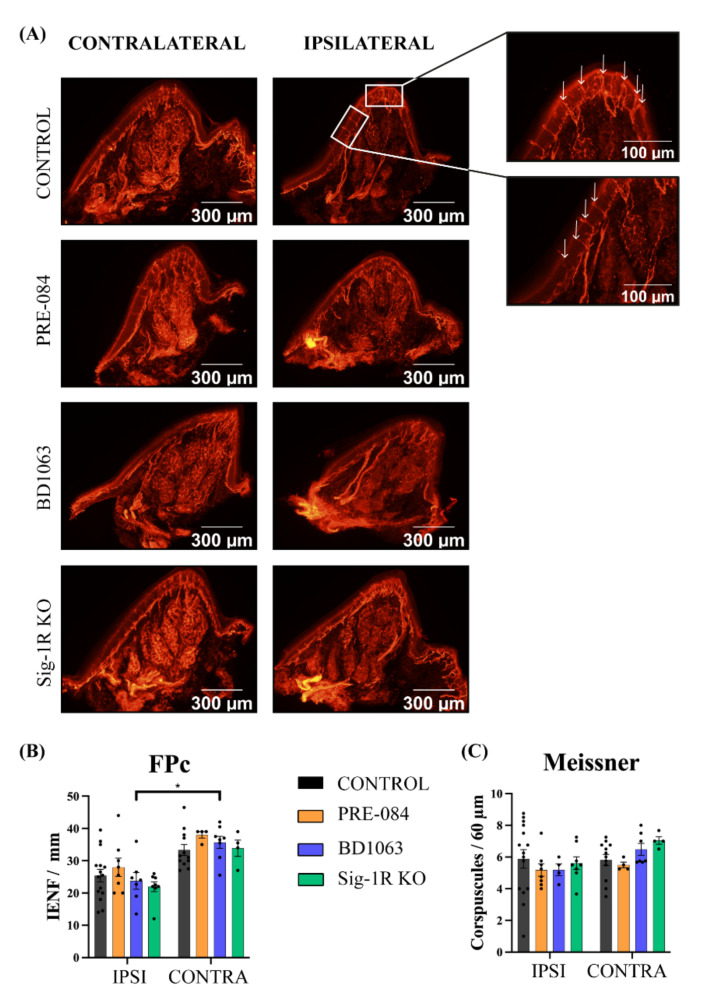
Reinnervation of intra-epidermal nerve fibers (IENF) and Meissner’s corpuscles. (**A**) Representative pictures (×10) of the C footpad immunolabeled for PGP9.5 in each group, ipsilateral and contralateral to the injury. Inset images showing Meissner’s corpuscles and IENF at the tip and at the side of the pad at higher magnification (×40). White arrows point to IENF. Histograms of the density of IENF (**B**) and the number of Meissner’s corpuscles (**C**) for each group in the study. Values are mean ± SEM, analyzed by two-way ANOVA and Bonferroni post-hoc test: * *p* < 0.05 ipsilateral footpad vs. the respective contralateral (n = 8 IPSI and n = 4 CONTRA for PRE-084 or Sig-1R KO groups; n = 7.4 IPSI and n = 8.7 CONTRA for BD1063; n = 15 IPSI and n = 12 CONTRA for control group).

To quantitatively assess the reinnervation of the paw skin by the regenerating axons, the IENF and the Meissner’s corpuscles of the footpads were analyzed by PGP9.5 immunolabeling (Figure 5A). Interestingly, the IENF number per length did not show differences between control untreated and treated animals, neither in the injured nor in the intact paw (Figure 5B). All the groups presented lower numbers of IENF at the ipsilateral footpad compared to the contralateral intact side, being statistically significant in the BD1063, control and Sig-1R KO groups. The number of Meissner’s corpuscles at the tip of the footpad did not show any difference (Figure 5C). Taken together, these data indicate good reinnervation of the denervated paw after 42 dpi, without a marked effect observed with either treatment with Sig-1R ligands or in the absence of this receptor.

**Figure 6:**
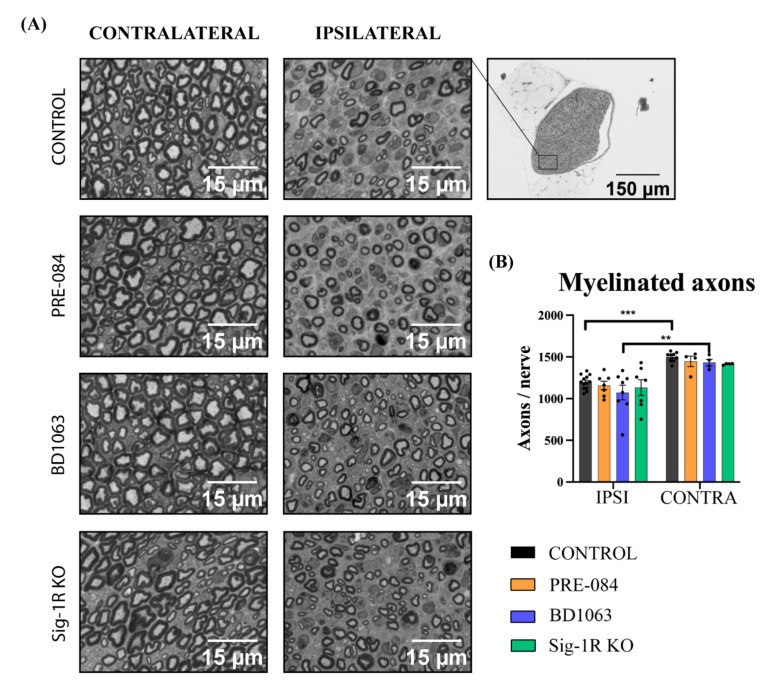
Myelinated axons in the regenerated tibial nerve. (**A**) Representative images (×100) of the tibial nerve in each group, ipsilateral (IPSI) and contralateral (CONTRA) to the injury; (**B**) Number of myelinated axons for each group in the studies of Sig-1R ligands and the Sig-1R KO mice. Values are mean ± SEM, analyzed by two-way ANOVA and Bonferroni post-hoc test: ** *p* < 0.01, *** *p* < 0.001 ipsilateral vs. respective contralateral nerve. PRE-084 or Sig-1R KO: n = 7 IPSI, n = 4 CONTRA; BD1063: n = 8 IPSI, n = 4 CONTRA; control: n = 13 IPSI, n = 9 CONTRA.

To directly assess the regeneration in the injured nerve, myelinated axons were counted in the distal tibial nerve. All the groups presented the typical morphology expected after a nerve injury, consisting of small myelinated axons, thin myelin sheaths and increased connective tissue between the axons (Figure 6A). The number of myelinated axons was significantly decreased in the regenerated tibial nerve compared to the intact nerve in all the experimental groups (Figure 6B). There were no differences between groups, indicating that no effects were induced either by Sig-1R ligands treatment or in Sig-1R KO mice.

### 3.5. BD1063 Treatment Reduces Infiltrating Macrophages in the Injured Nerve

**Figure 7:**
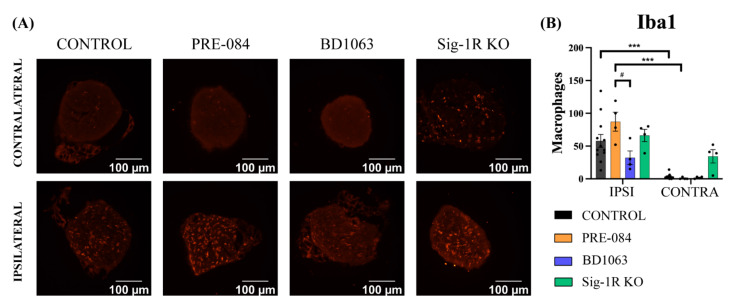
Macrophage infiltration in the regenerated nerve. (**A**) Representative pictures (×40) of the IBA1 immunolabeled cells in the regenerated and intact tibial nerves for each group; (**B**) Histograms of macrophage counting of the ipsilateral (IPSI) and contralateral (CONTRA) tibial nerves. Two-way ANOVA and Bonferroni post-hoc test: *** *p* < 0.001 injured nerve vs. the respective intact contralateral nerve; # *p* < 0.05 between injured nerve of PRE-084 and BD1063 treated groups (control: n = 12 IPSI, n = 11 CONTRA; rest of the groups: IPSI n = 4 and CONTRA n = 4).

To evaluate the inflammatory response in the regenerated nerve, the presence of macrophages was assessed by IBA1 immunostaining in the tibial nerve distal to the injury (Figure 7A). The number of macrophages was very low in all groups in the contralateral intact nerve (Figure 7B). On the other hand, in the regenerated nerves the number of infiltrating macrophages was markedly increased, even at the late time point that they were analyzed. Notably, there was an increased amount of IBA1+ cells in the tibial nerve of PRE-084 treated mice when compared to the control (not significant), and to the BD1063 group (*p* < 0.05). Interestingly, the Sig-1R KO mice showed a similar number of macrophages than the control group (Figure 7B).

## 4. Discussion

After a complete nerve transection, full functional recovery of the denervated regions is usually incomplete. Despite the fact that peripheral axons are able to regenerate, they need an appropriate tropic support, commonly consisting of the distal endoneurial matrix and the reactive Schwann cells. Thus, these injuries are usually managed by surgical repair in order to rejoin the sectioned nerve stumps. However, the rate of regeneration is slow, about 1–2 mm/day [1], and the chances for specific adequate regeneration and reinnervation are poor in transected, large nerves. The current work has evaluated a putative molecular target, the Sig-1R, in order to ascertain if its activation may enhance the rate of regeneration and improve functional recovery after PNI. The experimental model chosen was the complete transection and suture repair of the sciatic nerve, since it allows the evaluation of the timing and the amount of axonal regeneration using functional tests during follow-up and the histological final study, without confounding issues derived from collateral reinnervation that take place in partial nerve injury models [27]. The sciatic nerve cut and suture model in mice is characterized by the reinnervation of hind paw muscles and skin from about 21 days, therefore allowing an adequate time to study if the pharmacological treatment is able to increase the rate of nerve regeneration. The results obtained by treatment with two Sig-1R ligands, one considered as agonist (PRE-084) and one as antagonist (BD1063), show that they did not induce marked effects on the outcomes after sciatic nerve section and repair in mice. Furthermore, the Sig-1R KO mice showed a similar course of nerve regeneration than the corresponding WT mice.

It was previously reported that the modulation of Sig-1R produced neuroprotective and pro-regenerative effects [7,8,23,24,27]. In fact, the administration of Sig-1R agonist PRE-084 resulted in promoted neuritogenesis in in vitro studies [7,8]. Despite these previous findings, the overall outcome of the study indicates that PRE-084 treatment at 0.25 mg/kg produced only a slight advantage in the early stages of reinnervation, but not a significant improvement in the final levels of regeneration in the model used. The slight pro-regenerative activity of PRE-084 treatment was observed in terms of recovery of locomotor function (see Figure 2), and earlier recovery of CMAP and CNAP recordings (see Figure 3). The shorter motor and sensory latencies, observed at initial times of reinnervation, may correlate with previous findings regarding the increased expression of myelin basic protein (MBP) in Schwann cells when treated with PRE-084 [29]. However, to fully determine whether PRE-084 is a pro-regenerative agent in vivo, further studies at different concentrations should be conducted. In addition, the use of Sig-1R agonist treatment may be beneficial because of the well-demonstrated neuroprotective activity in more severe PNIs, after which a proportion of axotomized neurons die [22,23].

On the other hand, the Sig-1R antagonist BD1063 has been reported to counteract the PRE-084 effects in both in vitro [8] and in vivo [30] studies. However, Tsai and Su [11] have reported a positive effect of BD1063 administration in vitro on neurite elongation. Nevertheless, the results obtained in this study did not show any significant impact on regeneration of the BD1063 treatment in vivo. Interestingly, the results from the Sig-1R KO group also indicate that the lack of Sig-1R does not have any clear impact on peripheral nerve regeneration after injury.

Numerous studies have described the role of Sig-1R in the control of pain. In experimental models of pain, Sig-1R KO animals showed an attenuated pain response in the same line with animals treated with Sig-1R antagonists. Specifically, BD1063 treatment has been extensively studied in neuropathic pain models, causing analgesic effects [12,13,14,24]. The results of the Pincher test (see Figure 4B) indicated that mice treated with BD1063 had hyposensitivity after complete nerve injury and reinnervation, similar to what was observed in the Sig-1R KO group, corroborating previous studies with this compound. The contrary was observed in animals treated with the agonist PRE-084. In fact, increased mechanical allodynia in PRE-084 treated mice was observed after nociceptive system priming [31], therefore a similar effect is expected after a nerve injury that results in neuronal hyperexcitability and hyperalgesia in the reinnervated skin [27]. Interestingly, hypoalgesia was also found in the contralateral paw in the BD1063 group. This phenomenon could be explained by a systemic effect of the compound, suggesting an off-target or a triggered activity of Sig-1R in physiological state. Nevertheless, the assessment of IENF and Meissner’s corpuscles’ reinnervation suggests that Sig-1R did not affect the density of reinnervation, and most likely modulates the pathways of sensory transmission at the central nervous system.

On the other hand, it is known that nerve injury induces immune cell infiltration into the nerve during the Wallerian degeneration process and later in the axonal regeneration. IBA1 immunolabeling showed a tendency towards a higher number of macrophages in the regenerated tibial nerve of mice treated with PRE-084 after injury (see Figure 6). This correlates with previous findings after stroke in a rat model with SA4503 treatment, another Sig-1R agonist [32]. Contrarily, PRE-084 administration was reported to reduce microglial reactivity in the central nervous system in different neuropathological conditions related with neuronal death [22,30,33,34]. Moreover, the analgesic effect of Sig-1R antagonists for neuropathic pain has been related with a decreased glial reactivity after spinal cord injury [35,36] and other conditions of neuropathic pain [37]. In Sig-1R-KO mice the levels of macrophage/monocyte infiltration and pro-inflammatory cytokines in the DRG were found to be lower after nerve injury than in WT mice [21]. In fact, we observed reduced macrophage infiltration in the BD1063 group after sciatic nerve injury, correlating with the hypoalgesia found in the Pincher test. Similarly, the hypersensitivity observed in the PRE-084 group may correlate with the increased presence of macrophages. It is worth noting that the macrophage response was analyzed at a late time point, 42 dpi, well beyond the time of maximal macrophage infiltration, during the Wallerian degeneration phase. Therefore, it is not possible to preclude that the Sig-1R ligands may play a more noticeable role on the inflammatory response earlier after PNI.

In summary, pharmacological modulation of the Sig-1R, as well as its ablation, did not have marked effects on the process of nerve regeneration. However, this does not exclude the possibility that boosting its activity may be applicable for improving survival of axotomized neurons after more proximal injuries, such as at the spinal root level. Indeed, the slight improvements observed with PRE-084 treatment during the early phase of regeneration are of potential interest, and it may be worth conducting a dose-dependent study, as well as to assay other new Sig-1R agonists that may act on other pathways [38]. On the other hand, Sig-1R antagonist compounds appear as an adequate option as co-adjuvant treatment to prevent development of neuropathic pain, a condition of high prevalence after traumatic PNI.

## Data Availability

All data supporting the findings of this study are available from the authors under reasonable request.
